# Comparing Drugs for Out-of-hospital, Shock-refractory Cardiac Arrest: Systematic Review and Network Meta-analysis of Randomized Controlled Trials

**DOI:** 10.5811/westjem.2021.2.49590

**Published:** 2021-07-19

**Authors:** Karan Srisurapanont, Thachapon Thepchinda, Siriaran Kwangsukstith, Suchada Saetiao, Chayada Kasirawat, Worawan Janmayka, Wachira Wongtanasarasin

**Affiliations:** * Chiang Mai University, Faculty of Medicine, Chiang Mai, Thailand; † Chiang Mai University, Department of Emergency Medicine, Faculty of Medicine, Chiang Mai, Thailand

## Abstract

**Introduction:**

The benefit of medications used in out-of-hospital, shock-refractory cardiac arrest remains controversial. This study aims to compare the treatment outcomes of medications for out-of-hospital, shock-refractory ventricular fibrillation (VF) or pulseless ventricular tachycardia (pVT).

**Methods:**

The inclusion criteria were randomized controlled trials of participants older than eight years old who had atraumatic, out-of-hospital, shock-refractory VF/pVT in which at least one studied group received a medication. We conducted a database search on October 28, 2019, that included PubMed, Scopus, Web of Science, CINAHL Complete, and Cochrane CENTRAL. Citations of relevant meta-analyses were also searched. We performed frequentist network meta-analysis (NMA) to combine the comparisons. The outcomes were analyzed by using odds ratios (OR) and compared to placebo. The primary outcome was survival to hospital discharge. The secondary outcomes included the return of spontaneous circulation (ROSC), survival to hospital admission, and the neurological outcome at discharge. We ranked all outcomes using surface under the cumulative ranking score.

**Results:**

We included 18 studies with 6,582 participants. The NMA of 20 comparisons included 12 medications and placebo. Only norepinephrine showed a significant increase of ROSC (OR = 8.91, 95% confidence interval [CI], 1.88–42.29). Amiodarone significantly improved survival to hospital admission (OR = 1.53, 95% CI, 1.01–2.32). The ROSC and survival-to-hospital admission data were significantly heterogeneous with the I2 of 55.1% and 59.1%, respectively. This NMA satisfied the assumption of transitivity.

**Conclusion:**

No medication was associated with improved survival to hospital discharge from out-of-hospital, shock-refractory cardiac arrest. For the secondary outcomes, norepinephrine was associated with improved ROSC and amiodarone was associated with an increased likelihood of survival to hospital admission in the NMA.

## INTRODUCTION

Cardiac arrest remains one of the major causes of disability and mortality. The worldwide incidence of adult out-of-hospital cardiac arrest (OHCA) treated by emergency medical services was estimated at 62.3 per 100,000 person-years.^1^ However, the overall survival rate of OHCA is less than 10%.^2^ Four electrocardiographic rhythms in cardiac arrest include ventricular fibrillation (VF), ventricular tachycardia (VT), asystole, and pulseless electrical activity (PEA). According to the American Heart Association (AHA) guidelines, defibrillation is recommended for VF and pulseless VT (pVT). Shock-refractory VF/pVT is defined as VF or pVT resistant to one or more defibrillations.^3^,^4^ The AHA guidelines for Advanced Cardiac Life Support (ACLS) also recommend the use of epinephrine, amiodarone, and lidocaine after failing one or more defibrillations.^3^,^4^ However, due to the lack of compelling evidence, these agents are not strongly recommended (Class-IIb recommendations).^3^

The benefits of medications for refractory, shockable cardiac arrest remain controversial. In one network meta-analysis (NMA) of vasopressors, the combination of epinephrine, vasopressin, and methylprednisolone was associated with good neurological outcome at discharge and the return of spontaneous circulation (ROSC).^5^ While this NMA found no benefit of epinephrine, another meta-analysis showed an increased rate of ROSC and survival to hospital discharge for OHCA.^6^ Two NMAs of antiarrhythmic drugs found that lidocaine and amiodarone could improve the survival-to-hospital discharge rates of individuals with out-of-hospital, shock-refractory VF/pVT.^7^,^8^ In contrast, another meta-analysis found increased short-term and long-term survival with nifekalant, but not amiodarone treatment.^9^

To our knowledge, there has been no attempt to perform a NMA comparing different classes of medications for out-of-hospital, shock-refractory VF/pVT patients. The previous two NMAs only compared the benefit among antiarrhythmic drugs in those patients.^7^,^8^ Another NMA compared vasopressors in adults with both out-of-hospital and in-hospital cardiac arrest, but the subgroup analysis of shock-refractory VF/pVT was not explored.^5^ For these reasons, we conducted a NMA comparing the benefit of any medications in patients with out-of-hospital, shock-refractory VF/pVT.

## METHODS

This systematic review was reported in accordance with the “PRISMA Extension Statement for Reporting of Systematic Reviews Incorporating Network Meta-analyses of Health Care Interventions: Checklist and Explanations.”^10^ The protocol of this study was prospectively registered on the PROSPERO website (registration ID: CRD42020149976).

### Inclusion Criteria for a Trial

The inclusion criteria were as follows; 1) any randomized controlled trial (RCT) not applying a crossover design; 2) participants > 8 years old who had atraumatic, out-of-hospital, shock-refractory VF or pVT; 3) at least one studied group received a medication; and 4) a report of ROSC, survival to hospital admission, survival to hospital discharge, or neurological outcome at discharge. Good neurological outcome was defined by the cerebral performance category score of 1–2 or modified Rankin scale score of 0–3. The participants’ criteria were selected only from those aged eight or older because the pediatric cardiac arrest algorithm ends at eight years of age, and the automated external defibrillator can only be applied to those older than eight.

### Study Selection and Search Strategy

We performed a database search on October 28, 2019, that included PubMed, Scopus, Web of Science, Cochrane CENTRAL, Academic Search Complete, and CINAHL Complete. Citations from relevant meta-analyses were also searched.^5^,^8^ We searched the databases from their inceptions to the final search date, with no language limitation. The Medical Subject Headings terms included a combination of search terms with various spellings and endings: “shock-refractory,” “ventricular fibrillation,” “ventricular tachycardia,” “cardiac arrest’,” “heart arrest,” “cardiopulmonary resuscitation,” “prehospital,” and “out-of-hospital.” The detailed search terms are provided in the supplementary data (see Appendix). We collected the search results obtained from these databases and removed the duplicates. Non-duplicated citations were imported into the Rayyan QCRI website, and the abstracts of the citations were independently screened and selected by two authors (KS and TT). Any discrepancy was resolved by a consensus discussion.

### Data Extraction and Trial Quality Assessment

We designed a data extraction form to collect the age, eligible criteria, setting, gender, details of drug interventions, additional interventions, and the per-protocol outcomes (ROSC, survival to hospital admission, survival to hospital discharge, and neurological outcome at discharge). Three authors (KS, CK, and SS) independently extracted the data. The quality of the included study was also independently assessed by three authors (KS, SK, and WC) using the RoB2, a revised tool for assessing the risk of bias in randomized trials.^11^ The quality aspects assessed by this scale included randomization, deviations from the intended interventions, missing outcome data, measurement of the outcomes, and selection of reported results. Any discrepancy was resolved by a consensus discussion.

### Statistical Analysis

We estimated the odds ratios (OR) and their 95% confidence intervals (CI) of the outcome difference between each pair of intervention groups. The pairwise ORs were estimated using the following equation:

$$OR=\frac{events\;occurred\;in\;the\;first\;arm}{participants\;without\;an\;event\;in\;the\;first\;arm}\times \frac{participants\;without\;an\;event\;in\;the\;second\;arm}{total\;participants\;in\;the\;second\;arm}$$

An OR higher than one inferred the superior effect size of the first arm compared to the second arm. However, for the studies with more than two arms, we estimated the ORs in every pair of interventions. We excluded the analysis of interventions in addition to the randomized interventions, assuming that this study design could ameliorate the confounding effect.

We performed the frequentist NMA to compare the outcomes among the medications. Conventional meta-analysis provides a result from trials of head-to-head comparisons of two or more tests or interventions resulting in “direct evidence.” Thus, this issue makes it impossible to assess the relative treatment effect between comparators. A NMA helps to create an “indirect effect” when studies test interventions that have been compared with a common comparator but not directly against one another.^12^ The application of variance structure was determined by the levels of heterogeneity (fixed-effect model for I^2^ < 50% and random-effect model for I^2^ ≥ 50%). The NMA was conducted using the inverse variance method. And we ranked the outcomes by the surface under the cumulative ranking curve (SUCRA) method, the estimated summary result of treatment outcomes for ranking all of the competitive treatment, which is beneficial for a decision-making perspective, for example, selecting the treatment with the best credible evidence. The transitivity assumption of each NMA was evaluated by the node-splitting method. We used Egger’s test for funnel plot asymmetry to assess the publication bias. Any *P*-value of less than 0.05 was considered statistically significant.

We performed the NMA using the netmeta package in RStudio (RStudio, PBC, Boston, MA).^13^ The netmeta, netsplit, netrank, and funnel.netmeta functions were used for NMA, node-splitting analysis, the SUCRA score calculation, and the publication bias assessment, respectively.

## RESULTS

### Study Selection

**Figure 1:**
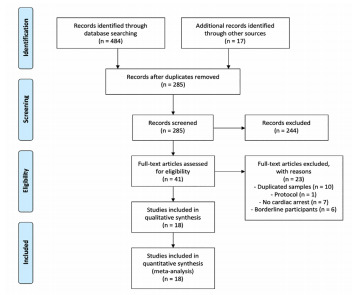
The PRISMA flow diagram.

**Figure 2:**
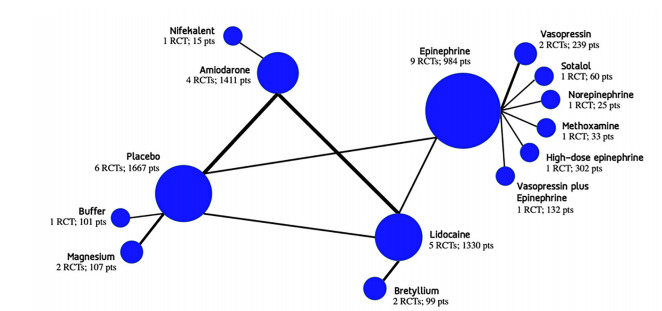
Network graph of 12 medications and placebo. The width of the lines is proportional to the sample size.

We found 501 relevant citations (Figure 1). After removing the duplicates, 285 citations remained. Of these, we excluded 244 articles by abstract screening, and an additional 23 articles were then excluded after full-text screening. In the conclusion. We included 18 studies with a total of 6,582 participants in this systematic review (see Appendix).^14^–^31^ This NMA compared 12 medications with placebo, which derived from 20 direct comparisons (Figure 2).

### Characteristics and Quality of the Included Studies

From all of the 18 included studies (Appendix), one study consisted of three experimental arms. Seven out of 10 antiarrhythmic drug trials administered epinephrine before the randomization. Out of 18 studies, 17 were conducted in Europe and America, while one was conducted in Japan. Participants were 60 years of age and older. The publication years of the trials ranged from 1981 to 2016. Non-pharmaceutical interventions, such as defibrillation and bystander Basic Life Support, were concurrently given in all trials. Using the RoB2, we found that 12 studies had a low risk of bias whereas the other six studies had some concerns.

### Survival to Hospital Discharge

**Figure 3:**
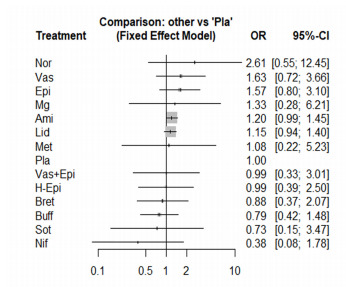
The forest plot of the network meta-analysis comparing the odds ratios on the survival to hospital discharge among the medications. *OR*, odds ratio; *CI*, confidence interval; *Ami*, amiodarone; Bret, bretylium tosylate; *Buff*, buffer; Epi,epinephrine; *H-Epi*, high-dose epinephrine; *Lid*, lidocaine; *Met*, methoxamine; *Mg*, magnesium sulfate; *Nif*, nifekalant; *Nor*, norepinephrine; *Pla*, placebo; *Sot*, sotalol; *Vas*, vasopressin.

The NMA of survival to hospital discharge consisted of 18 studies, including 20 pairwise comparisons. Because no significant heterogeneity was found (I^2^ = 0%) we conducted the NMA using a fixed-effect model. Among 13 medications compared to placebo, no medication significantly improved the survival to hospital discharge (Figure 3). Norepinephrine was the first ranking in survival to hospital discharge (SUCRA score = 0.85), followed by vasopressin (SUCRA score = 0.76) and epinephrine (SUCRA = 0.76). The head-to-head comparisons and the ORs of included medications are presented in the supplementary data. The NMA satisfied the assumption of transitivity as there was no significant difference between direct and indirect comparisons found by the node-splitting method. We did not find a significant publication bias using Egger’s test for funnel plot asymmetry (*P* = 0.46).

### Return of Spontaneous Circulation

**Figure 4:**
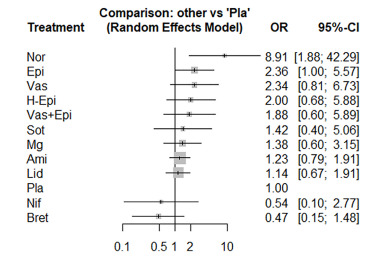
The forest plot of the network meta-analysis comparing the odds ratios on the return of spontaneous circulation among medications. *OR*, odds ratio; *CI*, confidence interval; *Ami*, amiodarone; *Bret*, bretylium tosylate; *Epi*, epinephrine; *H-Epi*, high-dose epinephrine; *Lid*, lidocaine; *Mg*, magnesium sulfate; *Nif*, nifekalant; *Nor*, norepinephrine; *Pla*, placebo; *Sot*, sotalol; *Vas*, vasopressin.

The NMA of ROSC consisted of 14 studies, including 16 pairwise comparisons. High heterogeneity was found (I^2^ = 55.1 %), so we conducted the NMA using a random-effect model. Among the 11 medications compared to placebo, only norepinephrine significantly improved the ROSC (OR = 8.91, 95% CI, 1.88–42.29) (Figure 4). Norepinephrine was also in the first ranking among the included medications (SUCRA score = 0.99), followed by epinephrine (SUCRA score = 0.76) and vasopressin (SUCRA score = 0.73). The head-to-head comparisons are presented in the supplementary data. The NMA satisfied the assumption of transitivity as there was no significant difference between direct and indirect comparisons found by the node-splitting method. Neither did we find a significant publication bias using Egger’s test for funnel plot asymmetry *(P* = 0.39).

### Survival to Hospital Admission

**Figure 5:**
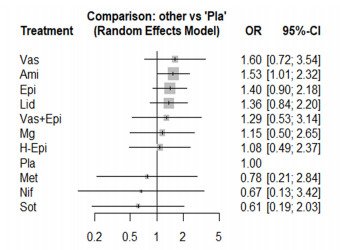
The forest plot of the network meta-analysis comparing the odds ratios on the survival to hospital admission among the medications. *OR*, odds ratio; *CI*, confidence interval; *Ami*, amiodarone; *Epi*, epinephrine; *H-Epi*, high-dose epinephrine; *Lid*, lidocaine; *Met*, methoxamine; *Mg*, magnesium sulfate; *Nif*, nifekalant; *Pla*, placebo; *Sot*, sotalol; *Vas*, vasopressin.

The NMA of survival to hospital admission consisted of 13 studies, including 18 pairwise comparisons. High heterogeneity was found (I^2^ = 59.1 %), so we conducted the NMA using a random-effect model. Among the 10 medications compared to placebo, only amiodarone (OR = 1.53, 95% CI, 1.01–2.32) significantly improved the survival to hospital admission (Figure 5). Moreover, amiodarone was the first ranking among the included medications (SUCRA score = 0.76), followed by vasopressin (SUCRA score = 0.75) and epinephrine (SUCRA score = 0.68). The head-to-head comparisons are presented in the supplementary data. The NMA satisfied the assumption of transitivity as there was no significant difference between direct and indirect comparisons found by the node-splitting method. Significant publication bias was found using Egger’s test for funnel plot asymmetry (*P* = 0.03).

### Good Neurological Outcome at Discharge

**Figure 6:**
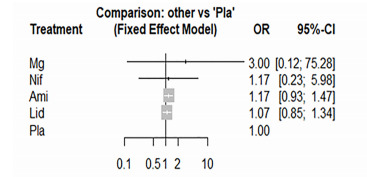
The forest plot of the network meta-analysis comparing the odds ratio of survival with good neurological outcomes among the pharmaceutical interventions. *OR*, odds ratio; *CI*, confidence interval; *Ami*, amiodarone; *Lid*, lidocaine; *Mg*, magnesium sulfate; *Nif*, nifekalant; *Pla*, placebo.

The NMA of good neurological outcome at discharge consisted of only three studies, including five pairwise comparisons. Heterogeneity analysis was not applicable due to the insufficiency of the data. No intervention could improve the neurological outcome at discharge (Figure 6). Magnesium sulfate was the first ranking among four medications (SUCRA score = 0.72). We could not perform the node-splitting method due to the data inadequacy. Publication bias analysis was also not appropriate due to the small number of included studies.

## DISCUSSION

We conducted this NMA to compare the treatment outcomes of multiple different medication classes in out-of-hospital, shock-refractory VF or pVT. This systematic review included moderate- and high-quality studies. The ROSC and survival to hospital admission data were highly heterogeneous. No medications improved survival to hospital discharge or neurological outcomes at discharge. Norepinephrine not only improved the ROSC but also demonstrate some benefit for survival to hospital discharge. Amiodarone was superior to placebo for the increased survival to hospital admission. All NMAs satisfied the assumption of transitivity. However, the publication bias of the survival to hospital admission might come from the fact that some studies did not report that outcome.

Despite comparing the different mechanisms of medication (antiarrhythmic drugs, vasopressors, steroid, etc.), NMA was a type of statistical approach designed to search for potential treatments that might not be directly compared. Besides, all NMAs in our study met the assumption of transitivity, which meant that potential treatment-effect modifiers were identified and balanced across the comparisons. The outcomes of antiarrhythmic drugs were inconsistent with the results of a previous NMA,^8^ as lidocaine and amiodarone were not associated with improved rates of survival to hospital discharge for out-of-hospital, shock-refractory VF or pVT. While a previous meta-analysis found benefit of nifekalant on short-term and long-term survival,^9^ the present NMA did not find its benefit on any outcomes.

These contrasting findings might be caused by the study designs of the included studies (the previous meta-analysis included RCTs, observational studies, and retrospective studies whereas our NMA included only RCTs). In contrast with the outcomes of antiarrhythmic drugs, the results of vasopressors were consistent with those of a previous NMA.^5^ A previous NMA did not find that norepinephrine significantly improved ROSC.^5^ Epinephrine was also inefficient for out-of-hospital, shock-refractory VF/pVT. Earlier evidence confirmed that epinephrine given within two minutes after the onset of shockable cardiac arrest decreased odds of ROSC and survival to hospital discharge.^32^ The current international guidelines for shockable, pulseless cardiac arrest recommend the use of epinephrine after the first defibrillation^4^; however, based on our findings epinephrine may not improve outcomes in this condition.

Current guidelines recommend two anti-arrhythmic agents for refractory, shockable cardiac arrest including amiodarone and lidocaine; however, a growing body of literature demonstrates the benefits of novel potential interventions – both pharmaceutical (ie, beta-blockers) and non-pharmaceutical (ie, switching pads location, double sequential defibrillation). Our included studies also included sotalol, which binds non-selectively to beta-adrenergic receptors. Nevertheless, sotalol did not exhibit positive effects in our study.

We propose three possible explanations for our findings. First, vasoconstriction may increase the likelihood of ROSC in those receiving cardiopulmonary resuscitation (CPR). Vasoconstriction increases coronary perfusion pressure (CPP) and myocardial blood flow, which have been posited as potential determinants of ROSC.^33^ However, nonspecific vasoconstriction may worsen post-resuscitation outcomes, which was consistent with our findings. One animal study demonstrated that endothelin-1, an intense vasoconstrictor, plus epinephrine improved CPP during CPR but had negative results in the post-resuscitation period.^34^ That norepinephrine, another powerful vasoconstrictor, improves ROSC would be supported by this explanation.

β2-adrenergic receptor agonists may be deleterious to shock-refractory VF/pVT. A preclinical study showed that β2- but not β1-adrenergic receptors increase calcium ion transients.^35^ As a result, the change in cytosolic calcium ion levels could perpetuate VF. β2-adrenergic receptor agonists, which have also been associated with cardiac arrest.^36^ This may explain why norepinephrine, which has predominate alpha receptor agonist, with lesser β1-agonism and no β2- agonism, was superior to epinephrine. Additionally, the benefits of amiodarone may further support this explanation as amiodarone is an antiarrhythmic drug with mild calcium channel blocker and beta-blocker properties. Its use during cardiac arrest as part of the current ACLS protocol could therefore ameliorate the β2-adrenergic effects induced by epinephrine.

As a third mechanism for medication effects, sodium channel activity has been associated with ventricular fibrillation. A preclinical study suggested that sodium channel activity could help maintain VF.^37^ The Na^+^ accumulated in the cytosolic can drive Ca^2+^ entry through the Na^+^-Ca^2+^ exchanger, and causes cytosolic and mitochondrial Ca^2+^ overload and eventual decline in myocardial function.^38^,^39^ This may explain why amiodarone and lidocaine, which are sodium channels blockers, could improve the outcomes of shock-refractory VF/pVT.

## LIMITATIONS

There are several limitations in our study. First, we encountered an insufficiency of data, especially for the neurological outcome at discharge. For example, the trial studying the treatment outcomes of norepinephrine did not provide the rate of survival to hospital admission and the neurological outcome at discharge.^26^ Second, the included trials were conducted in different years. As a result, ACLS algorithms and resuscitation qualities might vary among the studies. Third, because this NMA only compared the treatment outcomes of randomized drugs we could not take into account any add-on medication. Epinephrine was administered before the randomization of antiarrhythmic drugs in some studies. Thus, the treatment outcomes of amiodarone or lidocaine without epinephrine remains unknown.

Fourth, most comparisons had small sample sizes. Only one trial had more than 1000 participants.^24^ Moreover, the ROSC and survival to hospital admission were highly heterogeneous. Such heterogeneity might arise from the differences of additional treatments and the definitions of ROSC and survival to hospital admission. Furthermore, although norepinephrine demonstrated significant improvement in ROSC, only one study consisting of 50 participants in 1991 was included in the NMA, which resulted in an extremely wide range of CIs.^26^ Therefore, the results regarding norepinephrine might be inconcludable. Besides, the included trials had applied different protocols of intervention that might have resulted in variances in prehospital treatments among studies. Lastly, some comparisons in our NMA were not directly compared. So, these findings should be considered only as hypotheses. Large RCTs of direct comparisons are warranted to confirm the results.

## CONCLUSION

In this present study comparing different classes of agents administered during out-of-hospital, shock-refractory VF/pVT, no medication was associated with improved survival to hospital discharge. For the other outcomes, norepinephrine was associated with improved ROSC, and amiodarone was associated with an increased likelihood of survival to hospital admission in the NMA. Non-pharmaceutical interventions, such as defibrillation and bystander Basic Life Support, are still the mainstay treatment for this condition. Large, randomized controlled trials of medications for out-of-hospital, shock-refractory VF/pVT are warranted.

## Supplementary Information
