# Supplementary material for: Comparing Drugs for Out-of-hospital, Shock-refractory Cardiac Arrest: Systematic Review and Network Meta-analysis of Randomized Controlled Trials
Source: West J Emerg Med. 2021 Jul 19;22(4):834–41. doi: 10.5811/westjem.2021.2.49590 (PMC8328185; doi:10.5811/westjem.2021.2.49590)
Supplement: Supplementary file 1 [file wjem-22-834-s001.docx]

**Supplementary data**

*Appendix 1: Search terms (October 28, 2019)*

Pubmed: 151 citations

(shock-refractory OR shock-resistant OR refractory OR resist* OR persist* OR fail*) AND (VF OR VT OR pVT OR VF/pVT OR (ventricular fibrillation) OR (ventricular tachycardia) OR defibrillation OR shock*) AND ((cardiac OR cardiopulmonary OR circulat*) AND (arrest OR failure OR collapse OR resuscitation)) AND (out-of-hospital OR pre-hospital OR prehospital OR (outside hospital)) AND (random*)

Scopus: 32 citations

( TITLE-ABS-KEY ( ( shock-refractory  OR  shock-resistant  OR  refractory  OR  resist*  OR  persist*  OR  fail* ) )  AND  TITLE-ABS-KEY ( ( vf  OR  vt  OR  pvt  OR  vf/pvt  OR  ( ventricular  AND fibrillation )  OR  ( ventricular  AND tachycardia )  OR  defibrillation  OR  shock* ) )  AND  TITLE-ABS-KEY ( ( ( cardiac  OR  cardiopulmonary  OR  circulat* )  AND  ( arrest  OR  failure  OR  collapse  OR  resuscitation ) ) )  AND  TITLE-ABS-KEY ( ( out-of-hospital  OR  pre-hospital  OR  prehospital  OR  ( outside  AND hospital ) ) )  AND  TITLE-ABS-KEY ( ( random* ) ) )

Web of Science: 98 citations

TOPIC: ((shock-refractory OR shock-resistant OR refractory OR resist* OR persist* OR fail*) AND (VF OR VT OR pVT OR VF/pVT OR (ventricular fibrillation) OR (ventricular tachycardia) OR defibrillation OR shock*) AND ((cardiac OR cardiopulmonary OR circulat*) AND (arrest OR failure OR collapse OR resuscitation)) AND (out-of-hospital OR pre-hospital OR prehospital OR (outside hospital)) AND (random*))

Timespan: All years. Indexes: SCI-EXPANDED, SSCI, A&HCI, CPCI-S, CPCI-SSH, BKCI-S, BKCI-SSH, ESCI.

Cochrane CENTRAL: 111 citations

(shock-refractory OR shock-resistant OR refractory OR resist* OR persist* OR fail*) AND (VF OR VT OR pVT OR VF/pVT OR (ventricular fibrillation) OR (ventricular tachycardia) OR defibrillation OR shock*) AND ((cardiac OR cardiopulmonary OR circulat*) AND (arrest OR failure OR collapse OR resuscitation)) AND (out-of-hospital OR pre-hospital OR prehospital OR (outside hospital)) AND (random*) in Title Abstract Keyword

Academic Search Complete: 55 citations

(shock-refractory OR shock-resistant OR refractory OR resist* OR persist* OR fail*) AND (VF OR VT OR pVT OR VF/pVT OR (ventricular fibrillation) OR (ventricular tachycardia) OR defibrillation OR shock*) AND ((cardiac OR cardiopulmonary OR circulat*) AND (arrest OR failure OR collapse OR resuscitation)) AND (out-of-hospital OR pre-hospital OR prehospital OR (outside hospital)) AND (random*)

CINAHL Complete: 37 citations

(shock-refractory OR shock-resistant OR refractory OR resist* OR persist* OR fail*) AND (VF OR VT OR pVT OR VF/pVT OR (ventricular fibrillation) OR (ventricular tachycardia) OR defibrillation OR shock*) AND ((cardiac OR cardiopulmonary OR circulat*) AND (arrest OR failure OR collapse OR resuscitation)) AND (out-of-hospital OR pre-hospital OR prehospital OR (outside hospital)) AND (random*)

Hand Search: 17 citations

*Appendix 2:* Table of study characteristics

| **Study ID** | **Participants** | **Intervention arms** | **Guideline and additional interventions** |
| --- | --- | --- | --- |
| Allegra 2001 | Inclusion criteria   - 18 years old or older - Non-traumatic cardiac arrest presenting VF refractory to three electrical hocks | - 2g Epinephrine with either 2g Magnesium sulphate - 2g Epinephrine normal saline |  |
| Amino 2010 | Inclusion criteria   - Out-of-hospital cardiopulmonary arrest patients with first defibrillation failure or VF recurrence | - 125mg Amiodarone - 0.15mg/kg Nifekalant | CPR protocol of Tokai university |
| Dorian 2002 | Inclusion criteria   - Out-of-hospital ventricular fibrillation resistant to three shocks, intravenous epinephrine, and a further shock; or if they had recurrent ventricular fibrillation after initially successful defibrillation | - Amiodarone - Lidocaine | Guidelines 2000 for Cardiopulmonary Resuscitation and Emergency Cardiovascular Care |
| Dybvik 1995 | Inclusion criteria   - ≥ 16 years old - out-of-hospital cardiac arrest - persistent ventricular fibrillation after first defibrillation attempt or asystole   Exclusion criteria   - induced hypothermia - cardiac arrest of non-cardiac origin | Sodium bicarbonate 160 mmol/l, trometamol 300 mmol/l, disodium phosphate 20 mmol/l, and acetate 200 mmol/l  250 ml of 0.9% normal saline | Guidelines for cardiopulmonary resuscitation and emergency cardiac care 1992  Standards and Guidelines for Cardiopulmonary Resuscitation and Emergency Cardiac Care 1986  European Resuscitation Council Guidelines for advanced life support 1992 |
| Hassan 2002 | Inclusion criteria   - Patient who have either VF resistant to three defibrillatory shocks(refractory VF) or a second episode of VF during a resuscitation cycle for none VF treatment   Exclusion criteria   - Age less than 18 - Mechanism of CA being related to trauma,hanging or drowning | - 2g Magnesium sulphate + further 2g if the patient remained in VF after six defibrillations - placebo | ERC guideline |
| Haynes 1981 | Inclusion criteria   - Those whose rhythm became organized or remained in ventricular fibrillation   Exclusion criteria   - Patients whose rhythm converted to asystole or profound bradycardia with the initial shock(before administration of the study drug) | - Bretylium (500mg each) - Lidocaine (100mg each) |  |
| Kovoor 2005 | Inclusion criteria   - Patient with CARDIAC arrest due to refractory ventricular fibrillation   Exclusion criteria   - If asystole lasted >= 3 min preceding refractory ventricular fibrillation | - Sotalol 100 mg - Lignocaine 100 mg | Utstein Style guidelines |
| Kudenchuk 1999 | Inclusion criteria   - Adults with nontraumatic out-of-hospital cardiac arrest - Receive three or more precordial shocks still pulseless and had ventricular fibrillation or tachycardia. | - 1 mg epinephrine + 300 mg of amiodarone - 1 mg epinephrine + placebo | American Heart Association (AHA) Guidelines |
| Kudenchuk 2016 | Inclusion criteria   - 18 years or older - Nontraumatic out-of-hospital cardiac arrest and shock-refractory ventricular fibrillation or pulseless ventricular tachycardia - Intravenous or intraosseous vascular access   Exclusion criteria   - Patients who had already received open-label intravenous lidocaine or amiodarone during resuscitation or had known hypersensitivity to these drugs. | - 150 mg of amiodarone - 60 mg of lidocine | American Heart Association (AHA) Guidelines |
| Lindner 1991 | Inclusion criteria   - Out-of-hospital patients when 3 rapidly and consecutively administered direct-current shocks failed to convert VF | - 1 mg of Epinephrine - 1 mg of Norepinephrine | American Heart Association (AHA) Guidelines |
| Lindner 1997 | Inclusion criteria   - Patients in ventricular fibrillation resistant to electrical defibrillation | - Epinephrine (1 mg intravenously) - Vasopressin(40 U intravenously) | European Resuscitation Council and the American Heart Association |
| Olson 1984 | Exclusion criteria   - Pediatric patients ( those less than 18 years of age) - Patients who has traumatic PNBs - Poisoned patients | - Bretylium tosylate - Lidocaine | Milwaukee County Paramedic System |
| Olson 1989 | Inclusion criteria   - Patients in ventricular fibrillation not responding to initial defibrillations with a pulsatile rhythm | - Methoxamine (10 mg/ml) - Epinephrine (1 mg2ml) | The American Heart Association (AHA) protocol |
| Weaver 1990 | Inclusion criteria   - Patients with out-of-hospital cardiac arrest persisted in ventricular fibrillation after the first defibrillation attempt | - Lidocaine(100mg) - Epinephrine(0.5mg) | The American Heart Association (AHA) Guidelines |
| Wenzel 2004 | Inclusion criteria   - Out of hospital cardiac arrest - Ventricular fibrillation - Pulseless electrical activity - Asystole requiring CPR with vasopressor therapy   Exclusion criteria   - Successful defibrillation without the administration of a vasopressor - Documented terminal illness - Lack of intravenous access - Hemorrhagic shock - Pregnancy - Cardiac arrest after trauma - Age less than 18 | - Vasopressin 40 IU - Epinephrine 1 mg | The European agency for evaluation of medicinal product |

*Appendix 3:* Quality assessment of the included studies using the RoB2: A revised Cochrane risk-of-bias tool for randomized trials

RoB 2 is structured into 5 domains through which bias might be introduced into the result. These were identified based on both empirical evidence and theorical considerations. The five domains for individually randomized trials are: (1) Bias arising from the randomization process; (2) bias due to deviations from intended interventions; (3) bias due to missing outcome data; (4) bias in measurement of the outcome; and (5) bias in selection of the reported result.

| Study | Randomization | Deviation from the intended interventions | Missing outcome data | Measurement of outcome | Selection of the reported result | Overall |
| --- | --- | --- | --- | --- | --- | --- |
| Allegra 2001 | LR | LR | LR | LR | LR | LR |
| Amino 2010 | SC | LR | LR | LR | LR | SC |
| Dorian 2002 | LR | LR | LR | LR | LR | LR |
| Dybvik 1995 | LR | SC | LR | LR | LR | SC |
| Gueugniaud 1998 | LR | LR | LR | LR | LR | LR |
| Gueugniaud 2008 | LR | LR | LR | LR | LR | LR |
| Hassan 2002 | LR | LR | LR | LR | LR | LR |
| Haynes 1981 | LR | SC | LR | LR | LR | SC |
| Jacobs 2011 | LR | LR | LR | LR | LR | LR |
| Kovoor 2005 | LR | LR | LR | LR | LR | LR |
| Kudenchuk 1999 | LR | LR | LR | LR | LR | LR |
| Kudenchuk 2016 | LR | LR | LR | LR | LR | LR |
| Lindner 1991 | SC | SC | LR | LR | LR | SC |
| Lindner 1997 | LR | LR | LR | LR | LR | LR |
| Olson 1984 | SC | SC | LR | LR | LR | SC |
| Olson 1989 | SC | LR | LR | LR | LR | SC |
| Weaver 1990 | SC | SC | LR | LR | LR | SC |
| Wenzel 2004 | LR | LR | LR | LR | LR | LR |

LR: Low risk of bias; SC: Some concerns; HR: High risk of bias

*Appendix 4:* Abbreviations of pharmaceutical interventions

Ami = Amiodarone

Bret = Bretylium tosylate

Epi = Epinephrine

H-Epi = High-Dose Epinephrine

Lid = Lidocaine / Lignocaine

Mg = Magnesium Sulfate

Nif = Nifekalant

Nor= Norepinephrine

Pla = Placebo

Sot = Sotalol

Vas = Vasopressin

Met = Methoxamine

Buff = Buffer (bicarbonate)

*Appendix 5:* Return of Spontaneous Circulation

**Table 5S-1 Head-to-head odd ratio comparisons of the return of spontaneous circulation among the pharmaceutical interventions**

Drugs are reported in alphabetical order. Results are the ORs (with 95% CIs) in the column-defining treatment compared with the row-defining treatment. For efficacy, ORs higher than 1 favour the row-defining treatment.

|  | **Ami** | **Bret** | **Epi** | **H-Epi** | **Lid** | **Mg** | **Nif** | **Nor** | **Pla** | **Sot** | **Vas** | **Vas+Epi** |
| --- | --- | --- | --- | --- | --- | --- | --- | --- | --- | --- | --- | --- |
| **Ami** |  | 2.60  (0.85-8.00) | 0.52  (0.20-1.37) | 0.62  (0.19-1.97) | 1.08  (0.67-1.75) | 0.89  (0.35-2.27) | 2.29  (0.47-11.10) | 0.14  (0.03-0.70) | 1.23  (0.79-1.90) | 0.87  (0.23-1.91) | 0.53  (0.17-1.64) | 0.66  (0.19-2.23) |
| **Bret** |  |  | 0.20  (0.05-0.84) | 0.24  (0.05-1.14) | 0.42  (0.15-1.15) | 0.34  (0.08-1.40) | 0.88  (0.13-6.11) | 0.05  (0.1-0.37) | 0.47  (0.15-1.48) | 0.33  (0.06-1.48) | 0.20  (0.04-0.96) | 0.25  (0.05-1.27) |
| **Epi** |  |  |  | 1.18  (0.62-2.26) | 2.08  (0.76-5.68) | 1.71  (0.52-5.63) | 4.38  (0.69-27.88) | 0.26  (0.07-0.97) | 2.36  (1.00-5.57) | 1.66  (0.65-5.57) | 1.01  (0.55-1.86) | 1.26  (0.59-2.67) |
| **H-Epi** |  |  |  |  | 1.76  (0.53-5.83) | 1.45  (0.37-5.64) | 3.71  (0.52-26.42) | 0.22  (0.05-0.96) | 2.00  (0.68-5.88) | 1.41  (0.45-5.88) | 0.85  (0.35-2.09) | 1.07  (0.39-2.88) |
| **Lid** |  |  |  |  |  | 0.82  (0.31-2.18) | 2.11  (0.41-10.99) | 0.13  (0.03-0.66) | 1.14  (0.67-1.91) | 0.80  (0.20-1.91) | 0.49  (0.15-1.58) | 0.61  (0.17-2.13) |
| **Mg** |  |  |  |  |  |  | 2.56  (0.41-16.06) | 0.15  (0.03-0.90) | 1.38  (0.60-3.15) | 0.97  (0.21-3.15) | 0.59  (0.15-2.25) | 0.73  (0.18-3.01) |
| **Nif** |  |  |  |  |  |  |  | 0.06  (0.01-0.58) | 0.54  (0.10-2.77) | 0.38  (0.05-3.02) | 0.23  (0.03-1.62) | 0.29  (0.04-2.12) |
| **Nor** |  |  |  |  |  |  |  |  | 8.91  (1.88-42.29) | 6.28  (1.27-31.16) | 3.81  (0.91-16.01) | 4.75  (1.06-21.31) |
| **Pla** |  |  |  |  |  |  |  |  |  | 0.71  (0.20-2.52) | 0.43  (0.15-1.23) | 0.53  (0.17-1.67) |
| **Sot** |  |  |  |  |  |  |  |  |  |  | 0.60  (0.20-1.86) | 0.76  (0.22-2.51) |
| **Vas** |  |  |  |  |  |  |  |  |  |  |  | 1.25  (0.47-3.30) |
| **Vas+Epi** |  |  |  |  |  |  |  |  |  |  |  |  |

**Table 5S-2 Surface under the cumulative ranking (SUCRA) score ranking of return of spontaneous circulation among the pharmaceutical interventions**

|  | **P-score** |
| --- | --- |
| Norepinephrine | 0.9857 |
| Epinephrine | 0.7587 |
| Vasopressin | 0.7293 |
| High-dose Epinephrine | 0.6412 |
| Vasopressin plus Epinephrine | 0.6048 |
| Magnesium sulfate | 0.4856 |
| Sotalol | 0.4627 |
| Amiodarone | 0.4335 |
| Lidocaine | 0.3762 |
| Placebo | 0.2738 |
| Nifekalant | 0.1637 |
| Bretylium tosylate | 0.0849 |

**Table 5S-3 Node splitting method of the network meta-analysis of return of spontaneous circulation**

|  | k | Prop | NMA | Direct | Indirect | RoR | z | P-value |
| --- | --- | --- | --- | --- | --- | --- | --- | --- |
| Ami : Lid | 2 | 0.92 | 1.0836 | 1.0722 | 1.2257 | 0.8747 | -0.15 | 0.8825 |
| Ami : Pla | 2 | 0.96 | 1.2304 | 1.1498 | 5.7802 | 0.1989 | -1.45 | 0.1467 |
| Lid : Pla | 1 | 0.79 | 1.1355 | 1.2581 | 0.7763 | 1.6205 | 0.74 | 0.4586 |

k = number of studies

Prop = weight of direct effect size in the network meta-analysis

Direct = direct effect size

Indirect = indirect effect size

RoR = direct effect size to indirect effect size ratio

z = z-value of the difference between direct and indirect effect sizes

P-value = P-value of the difference between direct and indirect effect sizes


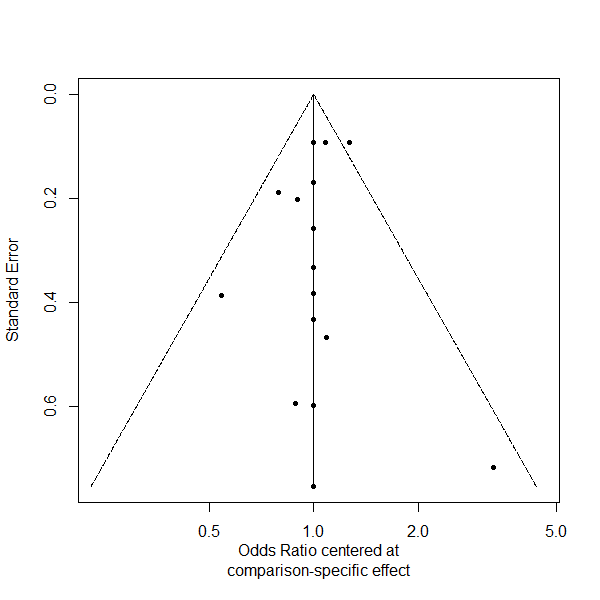


**Figure 5S-1: The funnel plot of the network meta-analysis comparing the return of spontaneous circulation among the pharmaceutical-intervention**


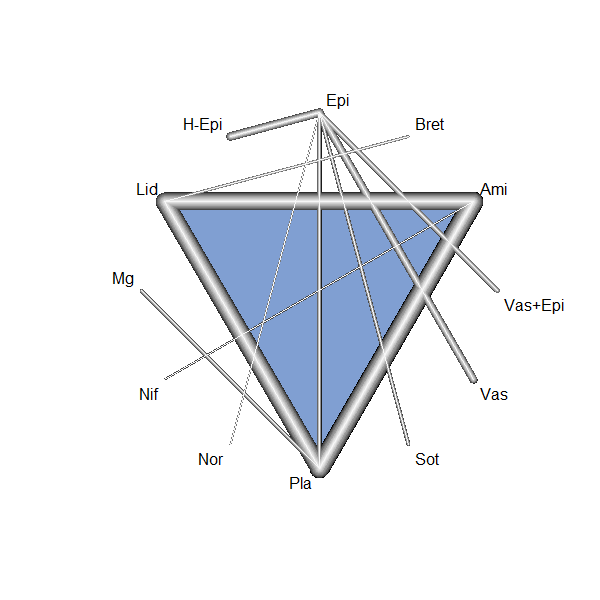


**Figure 5S-2: The network graph of the network meta-analysis comparing the return of spontaneous circulation among the pharmaceutical-intervention**


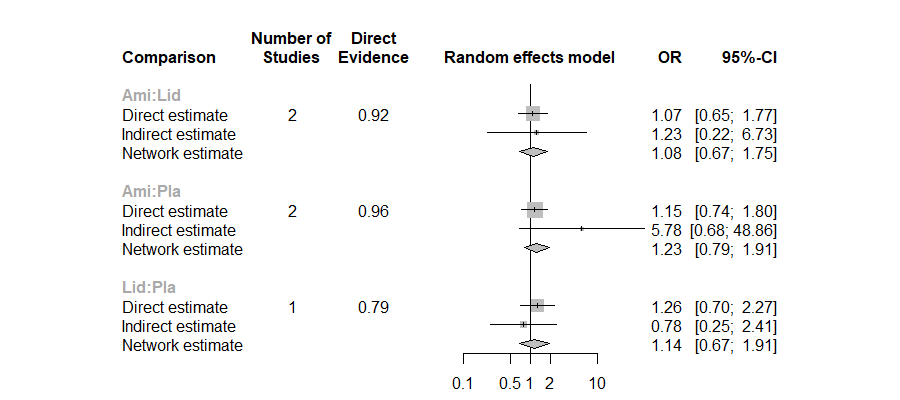


**Figure 5S-3: The node splitting graph of the network meta-analysis comparing the return of spontaneous circulation among the pharmaceutical-intervention**

*Appendix 6:* Survival to Hospital Admission

**Table 6S-1 Head-to-head odd ratio comparisons of the survival to hospital admission among the pharmaceutical interventions**

Drugs are reported in alphabetical order. Results are the ORs (with 95% CIs) in the column-defining treatment compared with the row-defining treatment. For efficacy, ORs higher than 1 favour the row-defining treatment.

|  | **Ami** | **Epi** | **H-Epi** | **Lid** | **Met** | **Mg** | **Nif** | **Pla** | **Sot** | **Vas** | **Vas+Epi** |
| --- | --- | --- | --- | --- | --- | --- | --- | --- | --- | --- | --- |
| **Ami** |  | 1.09  (0.67-1.75) | 1.41  (0.63-3.17) | 1.12  (0.69-1.81) | 1.97  (0.53-7.30) | 1.32  (0.52-3.37) | 2.28  (0.47-11.05) | 1.52  (1.00-2.32) | 2.49  (0.74-8.35) | 0.96  (0.42-2.16) | 1.19  (0.47-2.95) |
| **Epi** |  |  | 1.29  (0.67-2.49) | 1.03  (0.65-1.61) | 1.80  (0.53-6.12) | 1.21  (0.47-3.12) | 2.09  (0.40-10.88) | 1.40  (0.90-2.18) | 2.29  (0.75-6.94) | 0.88  (0.45-1.69) | 1.09  (0.50-2.37) |
| **H-Epi** |  |  |  | 0.79  (0.35-1.75) | 1.39  (0.34-5.55) | 0.93  (0.29-2.95) | 1.61  (0.27-9.48) | 1.07  (0.49-2.37) | 1.76  (0.48-6.38) | 0.68  (0.26-1.70) | 0.83  (0.30-2.31) |
| **Lid** |  |  |  |  | 1.75  (0.47-6.43) | 1.18  (0.45-3.08) | 2.03  (0.39-10.54) | 1.36  (0.84-2.19) | 2.22  (0.66-7.35) | 0.85  (0.38-1.89) | 1.06  (0.43-2.59) |
| **Met** |  |  |  |  |  | 0.67  (0.14-3.14) | 1.15  (0.14-8.99) | 0.78  (0.21-2.83) | 1.26  (0.24-6.58) | 0.49  (0.12-1.94) | 0.60  (0.14-2.56) |
| **Mg** |  |  |  |  |  |  | 1.72  (0.28-10.72) | 1.15  (0.50-2.64) | 1.87  (0.43-8.05) | 0.72  (0.22-2.27) | 0.89  (0.26-3.03) |
| **Nif** |  |  |  |  |  |  |  | 0.67  (0.13-3.41) | 1.09  (0.14-7.94) | 0.42  (0.07-2.46) | 0.52  (0.08-3.21) |
| **Pla** |  |  |  |  |  |  |  |  | 1.63  (0.49-5.39) | 0.63  (0.28-1.38) | 0.77  (0.31-1.89) |
| **Sot** |  |  |  |  |  |  |  |  |  | 0.38  (0.10-1.39) | 0.48  (0.12-1.85) |
| **Vas** |  |  |  |  |  |  |  |  |  |  | 1.24  (0.44-3.44) |
| **Vas+Epi** |  |  |  |  |  |  |  |  |  |  |  |

**Table 6S-2 Surface under the cumulative ranking (SUCRA) score ranking of survival to hospital admission among the pharmaceutical interventions**

|  | **P-score** |
| --- | --- |
| Amiodarone | 0.7554 |
| Vasopressin | 0.7499 |
| Epinephrine | 0.6798 |
| Vasopressin plus Epinephrine | 0.5840 |
| Magnesium sulfate | 0.5084 |
| High-dose Epinephrine | 0.4489 |
| Placebo | 0.3524 |
| Methoxamine | 0.3067 |
| Nifekalant | 0.2820 |
| Sotalol | 0.1918 |

**Table 6S-3 Node splitting method of the network meta-analysis of survival to hospital admission**

|  | k | Prop | NMA | Direct | Indirect | RoR | z | P-value |
| --- | --- | --- | --- | --- | --- | --- | --- | --- |
| Ami : Epi | 1 | 0.68 | 1.0900 | 1.2798 | 0.7746 | 1.6523 | 0.96 | 0.3353 |
| Ami : Lid | 2 | 0.83 | 1.1245 | 1.0519 | 1.5451 | 0.6808 | -0.60 | 0.5489 |
| Ami : Pla | 2 | 0.92 | 1.5297 | 1.3865 | 4.9887 | 0.2779 | -1.60 | 0.1092 |
| Epi : Lid | 2 | 0.91 | 1.0317 | 0.9180 | 3.3026 | 0.2780 | -1.60 | 0.1092 |
| Epi : Pla | 2 | 0.86 | 1.4034 | 1.3276 | 1.9510 | 0.6804 | -0.60 | 0.5485 |
| Lid : Pla | 1 | 0.69 | 1.3603 | 1.3508 | 1.3815 | 0.9778 | -0.04 | 0.9660 |

k = number of studies

Prop = weight of direct effect size in the network meta-analysis

Direct = direct effect size

Indirect = indirect effect size

RoR = direct effect size to indirect effect size ratio

z = z-value of the difference between direct and indirect effect sizes

P-value = P-value of the difference between direct and indirect effect sizes

**
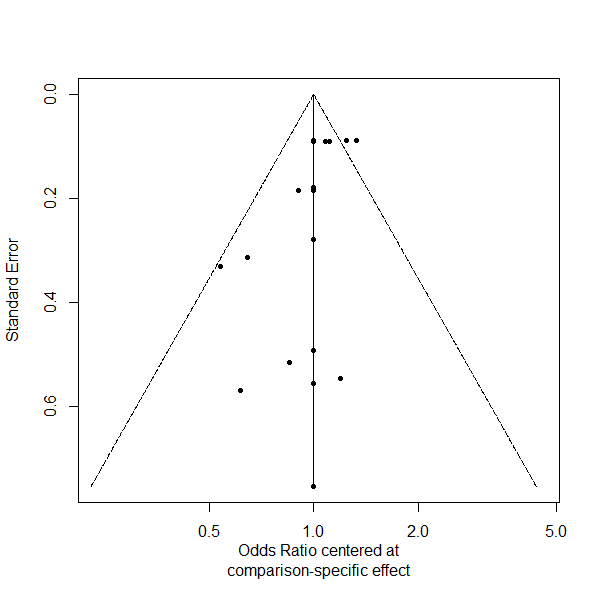
**

**Figure 6S-1: The funnel plot of the network meta-analysis comparing the survival to hospital admission among the pharmaceutical-intervention**


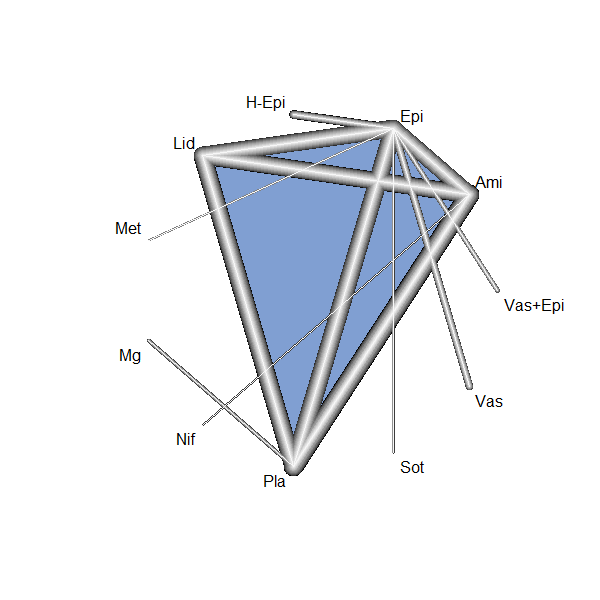


**Figure 6S-2: The network graph of the network meta-analysis comparing the survival to hospital admission among the pharmaceutical-intervention**


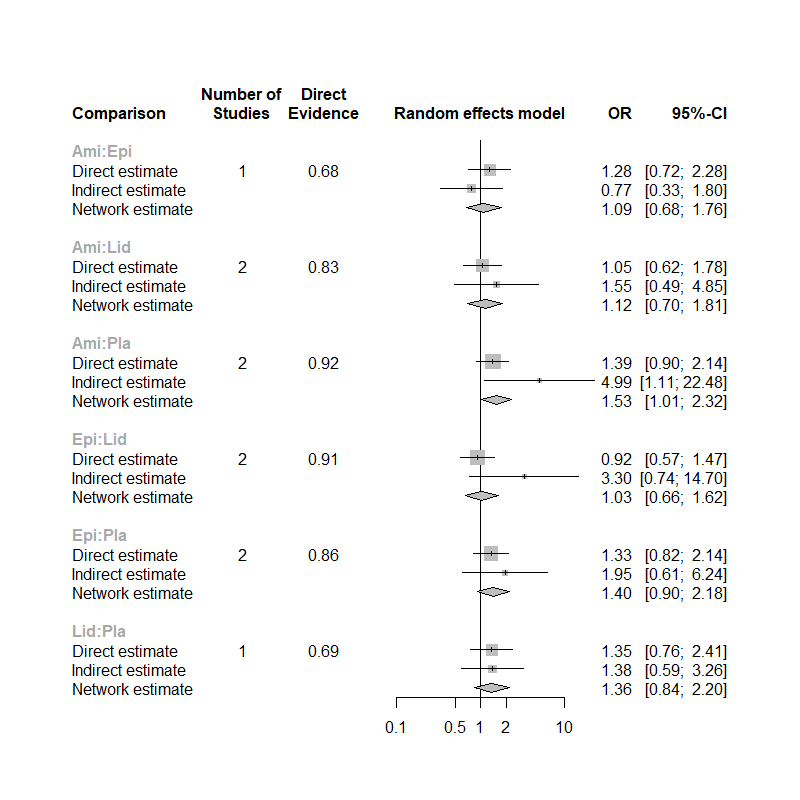


**Figure 6S-3: The node splitting graph of the network meta-analysis comparing the survival to hospital admission among the pharmaceutical-intervention**

*Appendix 7*: Survival to Hospital Discharge

**Table 7S-1 Head-to-head odd ratio comparisons of the survival to hospital discharge among the pharmaceutical interventions**

Drugs are reported in alphabetical order. Results are the ORs (with 95% CIs) in the column-defining treatment compared with the row-defining treatment. For efficacy, ORs higher than 1 favour the row-defining treatment.

|  | **Ami** | **Bret** | **Buff** | **Epi** | **H-Epi** | **Lid** | **Met** | **Mg** | **Nif** | **Nor** | **Pla** | **Sot** | **Vas** | **Vas+Epi** |
| --- | --- | --- | --- | --- | --- | --- | --- | --- | --- | --- | --- | --- | --- | --- |
| **Ami** |  | 1.36  (0.57-3.19) | 1.52  (0.78-2.94) | 0.76  (0.38-1.51) | 1.21  (0.47-3.08) | 1.04  (0.85-1.27) | 1.10  (0.22-5.34) | 0.90  (0.19-4.27) | 3.14  (0.68-14.50) | 0.46  (0.09-2.20) | 1.19  (0.98-1.45) | 1.63  (0.34-7.77) | 0.73  (0.32-1.67) | 1.20  (0.39-3.66) |
| **Bret** |  |  | 1.12  (0.38-3.23) | 0.55  (0.19-1.62) | 0.89  (0.25-3.07) | 0.77  (0.33-1.75) | 0.81  (0.13-4.78) | 0.66  (0.11-3.86) | 2.31  (0.40-13.30) | 0.34  (0.05-1.97) | 0.88  (0.37-2.06) | 1.20  (0.20-6.97) | 0.54  (0.17-1.72) | 0.89  (0.22-3.51) |
| **Buff** |  |  |  | 0.50  (0.19-1.26) | 0.79  (0.25-2.45) | 0.69  (0.35-1.33) | 0.73  (0.13-3.95) | 0.59  (0.11-3.14) | 2.07  (0.39-10.93) | 0.30  (0.05-1.63) | 0.78  (0.41-1.48) | 1.07  (0.20-5.76) | 0.48  (0.17-1.35) | 0.79  (0.22-2.83) |
| **Epi** |  |  |  |  | 1.59  (0.84-3.00) | 1.37  (0.70-2.67) | 1.45  (0.35-5.99) | 1.18  (0.21-6.38) | 4.13  (0.77-22.05) | 0.60  (0.14-2.46) | 1.57  (0.79-3.09) | 2.14  (0.52-8.69) | 0.97  (0.61-1.51) | 1.58  (0.65-3.79) |
| **H-Epi** |  |  |  |  |  | 0.86  (0.34-2.16) | 0.91  (0.19-4.31) | 0.74  (0.12-4.50) | 2.59  (0.43-15.57) | 0.38  (0.08-1.77) | 0.99  (0.39-2.50) | 1.35  (0.29-6.26) | 0.61  (0.28-1.32) | 0.99  (0.34-2.92) |
| **Lid** |  |  |  |  |  |  | 1.06  (0.22-5.08) | 0.86  (0.18-4.09) | 3.01  (0.64-14.07) | 0.44  (0.09-2.09) | 1.15  (0.93-1.40) | 1.57  (0.33-7.38) | 0.71  (0.32-1.58) | 1.15  (0.38-3.47) |
| **Met** |  |  |  |  |  |  |  | 0.82  (0.09-7.39) | 2.84  (0.31-25.59) | 0.42  (0.05-3.07) | 1.08  (0.22-5.22) | 1.48  (0.20-10.85) | 0.67  (0.15-2.95) | 1.09  (0.20-5.77) |
| **Mg** |  |  |  |  |  |  |  |  | 3.48  (0.39-30.84) | 0.51  (0.05-4.58) | 1.33  (0.28-6.21) | 1.81 (0.20-16.20) | 0.81  (0.14-4.67) | 1.33  (0.20-8.91) |
| **Nif** |  |  |  |  |  |  |  |  |  | 0.15  (0.01-1.30) | 0.38  (0.08-1.78) | 0.52  (0.05-4.61) | 0.23  (0.04-1.33) | 0.38  (0.06-2.53) |
| **Nor** |  |  |  |  |  |  |  |  |  |  | 2.60  (0.54-12.45) | 3.56  (0.48-25.91) | 1.60  (0.36-7.04) | 2.62  (0.49-13.76) |
| **Pla** |  |  |  |  |  |  |  |  |  |  |  | 1.36  (0.28-6.46) | 0.62  (0.27-1.38) | 1.01  (0.33-3.04) |
| **Sot** |  |  |  |  |  |  |  |  |  |  |  |  | 0.45  (0.10- 1.96) | 0.74  (0.14-3.83) |
| **Vas** |  |  |  |  |  |  |  |  |  |  |  |  |  | 1.63  (0.61-4.36) |
| **Vas+Epi** |  |  |  |  |  |  |  |  |  |  |  |  |  |  |

| **Table 7S-2 Surface under the cumulative ranking (SUCRA) score ranking of survival to hospital discharge among the pharmaceutical interventions** |
| --- |
| \|  \| \| --- \|  \|  \| **P-score** \| \| --- \| --- \| \| Norepinephrine \| 0.8471 \| \| Vasopressin \| 0.7588 \| \| Epinephrine \| 0.7562 \| \| Amiodarone \| 0.6034 \| \| Magnesium sulfate \| 0.5895 \| \| Lidocaine \| 0.5533 \| \| Methoxamine \| 0.4988 \| \| Vasopressin plus Epinephrine \| 0.4420 \| \| High-dose Epinephrine \| 0.4268 \| \| Placebo \| 0.4011 \| \| Bretylium \| 0.3750 \| \| Sotalol \| 0.3276 \| \| Buffer \| 0.2920 \| \| Nifekalant \| 0.1284 \| |

**Table 7S-3 Node splitting method of the network meta-analysis of survival to hospital discharge**

|  | k | Prop | NMA | Direct | Indirect | RoR | z | P-value |
| --- | --- | --- | --- | --- | --- | --- | --- | --- |
| Ami : Lid | 2 | 0.96 | 1.0436 | 1.0609 | 0.7021 | 1.5110 | 0.79 | 0.4297 |
| Ami : Pla | 2 | 0.99 | 1.1981 | 1.1853 | 2.8829 | 0.4111 | -0.99 | 0.3214 |
| Epi : Lid | 1 | 0.66 | 1.3698 | 1.2095 | 1.7379 | 0.6959 | -0.50 | 0.6140 |
| Epi : Pla | 1 | 0.36 | 1.5725 | 1.9800 | 1.3379 | 1.4369 | 0.50 | 0.6140 |
| Lid : Pla | 1 | 0.93 | 1.1480 | 1.1640 | 0.9416 | 1.2362 | 0.51 | 0.6104 |

k = number of studies

Prop = weight of direct effect size in the network meta-analysis

Direct = direct effect size

Indirect = indirect effect size

RoR = direct effect size to indirect effect size ratio

z = z-value of the difference between direct and indirect effect sizes

P-value = P-value of the difference between direct and indirect effect sizes


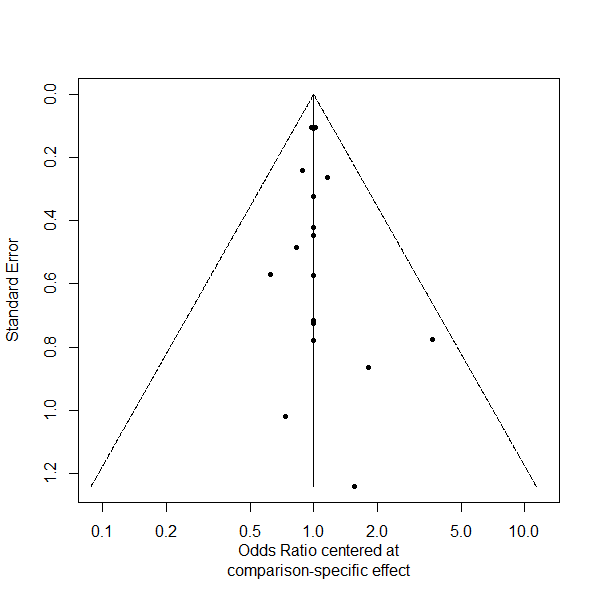


**Figure 7S-1: The funnel plot of the network meta-analysis comparing the survival to hospital discharge among the pharmaceutical-intervention**


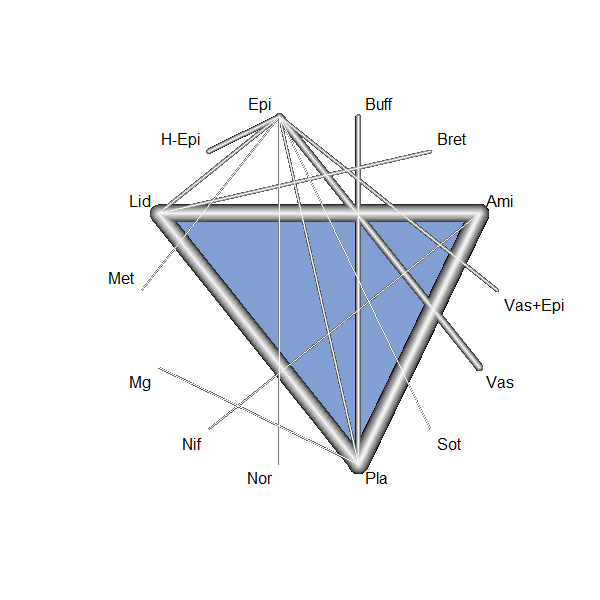


**Figure 7S-2: The network graph of the network meta-analysis comparing the survival to hospital discharge among the pharmaceutical-intervention**


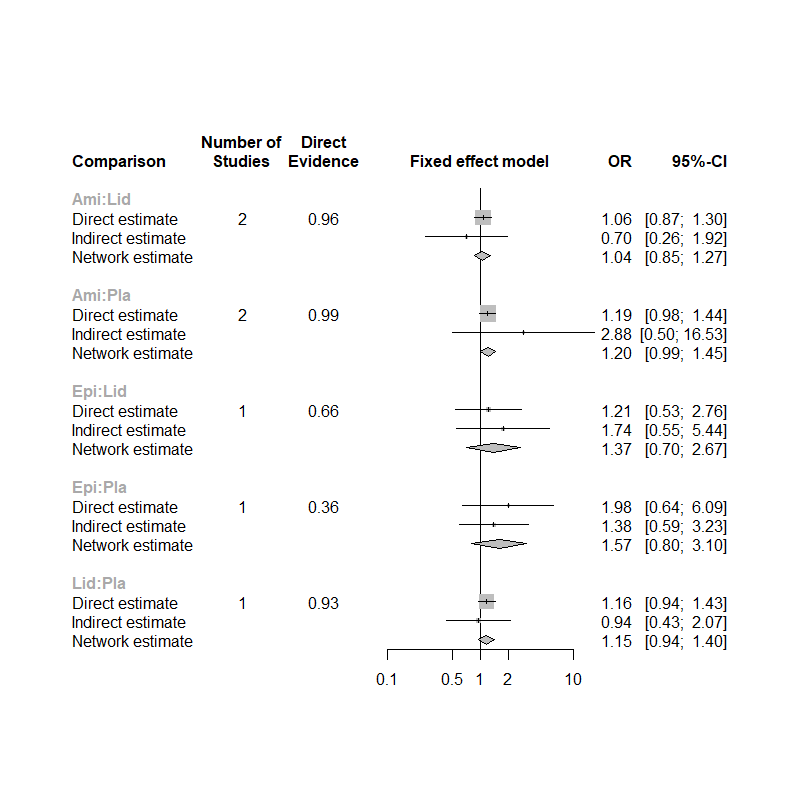


**Figure 7S-3: The node splitting graph of the network meta-analysis comparing the survival to hospital discharge among the pharmaceutical-intervention**

*Appendix 8*: Survival with Good Neurological Outcome

**Table 8S-1 Head-to-head odd ratio comparisons of the survival with good neurological outcome among the pharmaceutical interventions**

|  | **Ami** | **Lid** | **Mg** | **Nif** | **Pla** |
| --- | --- | --- | --- | --- | --- |
| **Ami** |  | 1.09  (0.86-1.37) | 0.39  (0.02-9.83) | 1.00  (0.19-5.04) | 1.16  (0.92-1.46) |
| **Lid** |  |  | 0.36  (0.01-8.98) | 0.91  (0.17-4.68) | 1.07  (0.84-1.34) |
| **Mg** |  |  |  | 2.57  (0.06-95.44) | 3.00  (0.11-75.27) |
| **Nif** |  |  |  |  | 1.17  (0.22-5.97) |
| **Pla** |  |  |  |  |  |

Drugs are reported in alphabetical order. Results are the ORs (with 95% CIs) in the column-defining treatment compared with the row-defining treatment. For efficacy, ORs higher than 1 favour the row-defining treatment.

**Table 8S-2 SUCRA score ranking of survival with good neurological outcome among the pharmaceutical interventions**

|  | P-score |
| --- | --- |
| Magnesium | 0.7239 |
| Amiodarone | 0.6170 |
| Nifekalant | 0.4801 |
| Lidocaine | 0.4117 |
| Placebo | 0.2674 |
